# High Tech POCUS Education in Remote Environments: An App Review

**DOI:** 10.24908/pocus.v8i2.16780

**Published:** 2023-11-27

**Authors:** Jeremy J Webb, Chad Mosby, John Stadnyk, Michael Jones

**Affiliations:** 1 Emergency Medicine Residency Program, LewisGale Medical Center Salem, VA USA

**Keywords:** Medical Education, Wilderness Medicine, Residency training, Technology in Education

## Letter

In recent years, the development of hand-held devices have intrigued POCUS enthusiasts due to improved affordability, portability, and ease of use. They also provide extra functionality for image storage and transmission for remote provider-to-provider communication and review. Due to these capabilities, portable ultrasound has found its use expanded to pre-hospital, wilderness, and austere settings, where cart-based machines and other imaging modalities are not an option 1. As resident educators, it is exciting to see the enthusiasm our trainees have for POCUS, prehospital, and wilderness medicine. But how do we train the next generation of POCUS wielders for best use in remote environments?

**Figure 1:**
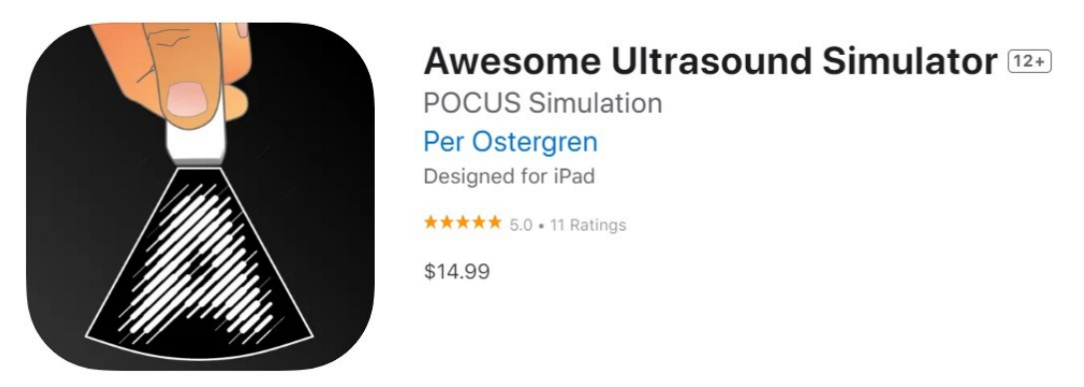
The Awesome Ultrasound Simulator App may be found on the iOS app store.

**Figure 2:**
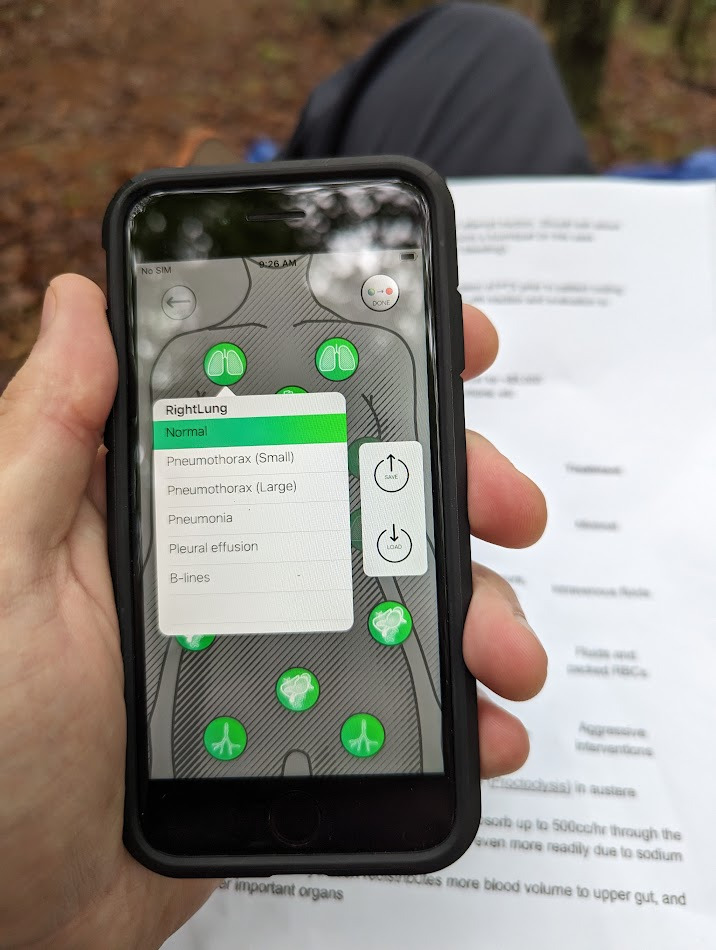
The “Remote” device (iPhoneTM) may be used to trigger the display of normal or pathologic cine loops.

**Figure 3:**
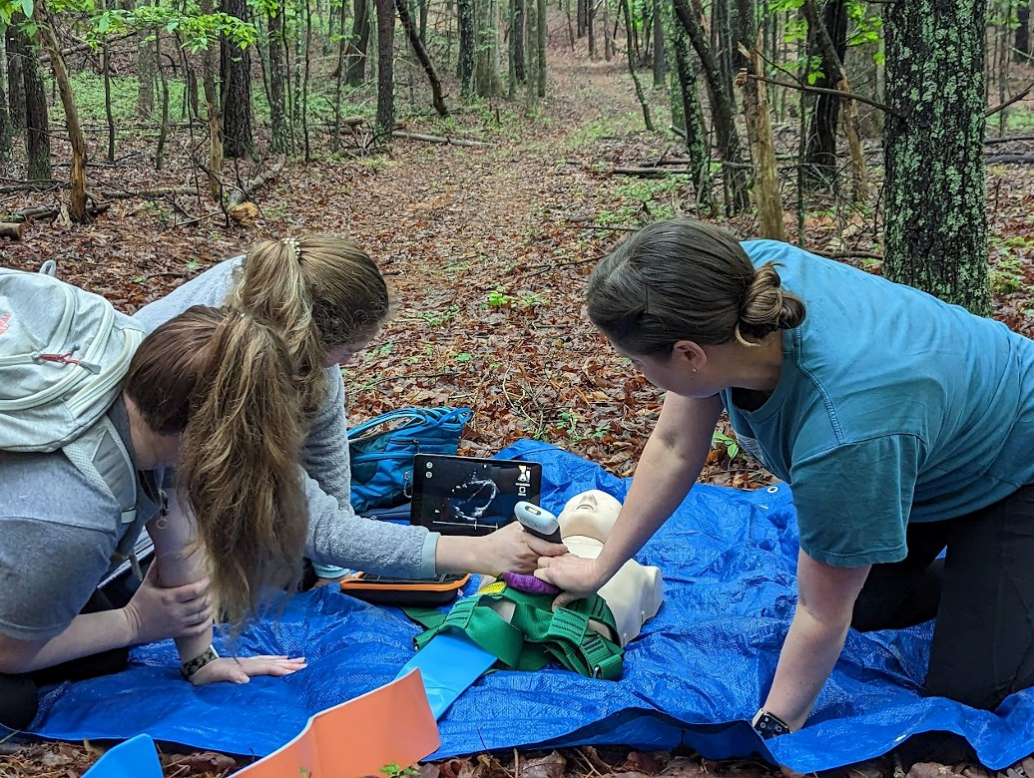
Emergency Medicine residents performing an E-FAST exam in the field, interpreting images on the “Monitor” device (iPadTM). An unconnected Clarius handheld device is used as the dummy probe.

Queue the “Awesome Ultrasound Simulator”, an iOS app created by Swedish physician Per Östergren (Figure 1) 2. Advertising a method to “Train as you fight, and fight as you train”, the app boasts a unique way to teach POCUS image acquisition and image interpretation in any environment. Currently, it costs $14.99 USD and its use requires two iOS devices. One serves as the “remote” and the other as the “monitor”. There are several cases to choose from, with preloaded cine loops, but there is also the ability to upload your own clips as well. The student may use a dummy probe or an untethered hand-held ultrasound device to phantom-scan either a manikin or a live person. The instructor can then wirelessly trigger cine loops via the “remote” device to the “monitor” device based on the site scanned for the student to interpret (Figures 2 and 3).

We recently had the opportunity to trial this app during our residency’s annual Wilderness Medicine simulation day. The event was held outside of town, at the foot of the Blue Ridge Mountains, on a plot of land with lots of acreage and wooded forest. In addition to map and topography assessment, improvised splinting, field extrication, and tourniquet application, we included a station requiring use of a hand-held ultrasound device to clinch a diagnosis. Our scenario involved a hunter (manikin) who had fallen out of a tree-stand from a significant height with resultant chest wall trauma. Residents were required to perform a trauma survey and identify a pneumothorax through identification of lack of lung sliding and a lung point on simulated POCUS assessment. Not only could residents phantom-scan the chest wall and lung, but they could perform a brief cardiac exam and a FAST examination as well. 

The Awesome Ultrasound Simulator worked as advertised and provided a realistic feel to the scenario. There is a small learning curve for uploading clips to the device, and when using in a remote location you must ensure adequate battery charges on your devices. The app leans heavy on image interpretation over image acquisition, although instructors may withhold triggering of a cine loop until appropriate probe positioning by the trainee occurs. All in all, our department found tremendous use in this easy to use and affordable application. The ability to simulate real pathology and promote out-of-hospital use of POCUS was both impressive and fun. We hope to continue to use the app in efforts to teach high tech diagnosis in remote environments, and maybe you will too. 

## Disclosures

The authors have no conflicts of interest to share. We share no relationship, financial or otherwise, with the “Awesome Ultrasound Simulator” application or its creator. The opinions above are our own.
